# Systems Thinking, Causal Loop Diagram, and Systems Dynamic in Public Health Challenges: Navigating Long COVID Syndrome and Sense of Smell in LGBTQIA+ Communities

**DOI:** 10.1002/puh2.70004

**Published:** 2024-09-03

**Authors:** Behnaz Akbari, Jessica M. Wang, Namdar Baghaei‐Yazdi, Hooshang Lahooti, Janet Hope Sherman

**Affiliations:** ^1^ Department of Chemistry Purdue University West Lafayette Indiana USA; ^2^ College of Liberal Arts & Sciences School of Life Sciences University of Westminster London UK; ^3^ Sydney Medical School University of Sydney Sydney Australia; ^4^ Medical School Notre Dame University Sydney Australia; ^5^ Chobanian & Avedisian School of Medicine Boston University Boston Massachusetts USA

**Keywords:** LGBTQIA+, long COVID, public health, smell loss, systems thinking

## Abstract

**Background:**

The coronavirus pandemic has profoundly affected global health, economic stability, and environmental sustainability. Despite these challenges, significant gaps in data remain, particularly in effectively assessing and engaging diverse communities such as color, LGBTQIA+ individuals, and low‐income groups. This shortage of comprehensive research limits our capacity to undertake sensitive studies, specifically in dealing with the complexities of long COVID, which some individuals continue to suffer from after their initial recovery.

**Objective:**

This review delves into the ongoing repercussions of long‐term COVID‐19, a postinfectious syndrome marked by neurological symptoms such as cognitive deficits and sensory impairments, which may last well beyond the acute phase of the illness. These symptoms frequently overlap with mental health issues (e.g., anxiety and depression), which can aggravate the socioeconomic challenges faced by vulnerable populations, especially within the LGBTQA+ communities.

**Methods:**

To tackle these complex interactions, we have introduced a novel public health framework: model‐based systems thinking (MBST), which incorporates System Dynamics and causal loop diagrams (CLD).

**Results and Discussion:**

The articles were selected on the basis of their discussion of COVID‐19‐associated anosmia, exploration of olfactory dysfunction alongside neurocognitive disorders, and the challenges experienced in LGBQA+ communities. This approach offers a robust framework for dissecting the intricate ties between socioeconomic factors, health outcomes, and the extended recovery trajectories associated with long‐term COVID‐19, with a particular focus on olfactory dysfunction. We also explore strategies to make our models more accessible to healthcare providers and the LGBTQA+ communities, encouraging its broader adoption.

**Conclusion:**

Long COVID's impact on public health and marginalized communities highlights the urgent need for adopting systems thinking models. Additionally, this article calls for a concerted effort from all experts to foster multidisciplinary, team‐based research and implement effective support measures for COVID‐19 survivors across all communities, mainly focusing on the scientific, social, and behavioral challenges LGBTQIA+ and low‐income individuals face.

## Graphical abstract

This graphical abstract illustrates the multifaceted approach to understanding and addressing long COVID‐19, highlighting the integration of natural remedies, diverse medical personnel, and the impacts on public health and LGBTQ+ communities, reflecting the interconnectedness of biological, social, and healthcare dimensions.

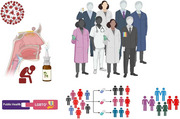

## 1 Introduction

Long coronavirus disease is a severe and complex public health challenge that can result from a previous SARS‐CoV‐2 infection. It encompasses a broad spectrum of persistent symptoms and health problems for weeks, months, or even years beyond the initial COVID‐19 [1, 2, 3]. The long‐term pathogenesis of COVID‐19 is believed to involve immune dysregulation, disturbances in the microbiota, autoimmunity, abnormalities in clotting and endothelial functions, and emotional distress, as highlighted in recent studies [2]. Individuals recovering from this illness may experience disruptions in their sleep and increased fatigue due to hormonal imbalances, proinflammatory biomarkers, and central nervous system impairment stemming from the infection [2, 3, 4, 5, 6]. Thus, this condition can be both debilitating and isolating, particularly in the absence of clear and immediate treatment solutions. In addition, long‐term COVID is characterized by recurring symptoms and inflammation across multiple organ systems, and diagnosis can be challenging, influenced by factors like demographics, comorbidities, and immune responses [7, 8]. Regardless of initial disease severity, almost 2 years after the initial COVID‐19 infection, survivors generally show improvements in both physical and mental health, with many returning to work. However, the prevalence of long‐term symptoms remains significant, and overall health status is notably lower compared to the general population [9]. A growing number of studies have shown that losing important senses such as smell and taste might have unfavorable consequences on the quality of life of COVID‐19 survivors [6, 10–14]. Olfactory dysfunction has various implications and consequences, ranging from difficulty detecting dangerous pathogens to hindering social functioning and behaviors [15, 16]. Those who lost their sense of smell affected their physical health, work life, sleeping quality, partnership, emotional stability, and leisure [16, 17, 18, 19, 20, 21].

Health disparities, particularly related to marginalized communities, contribute to a higher burden of infection and ongoing symptoms in these minority populations—communities that often encounter social, political, and economic exclusion and discrimination [22]. Consequences of marginalization cause various adverse outcomes like poverty, poor health outcomes, violence, and systemic inequality, which perpetuate the cycle of marginalization. COVID‐19 has contributed to a worsening of life circumstances (e.g., physical health, mental health, financial stability, meeting basic needs, and feelings of social connectedness) among the LGBTQIA+ community (the acronym LGBTQIA+ stands for lesbian, gay, bisexual, transgender, queer, intersex, and asexual, representing various sexual orientations and gender identities; the “+” symbol includes additional identities not explicitly covered by the initial letters) with specific outcomes such as anxiety and depression [3, 23, 24].

Systems Thinking provides a dynamic and practical framework that enables scientists from various disciplines to tackle the complex challenges of long COVID across all populations, particularly within the LGBTQIA+ community, during and beyond the COVID‐19 pandemic. For instance, a qualitative evidence synthesis conducted by Lucas et al. identified several categories of COVID‐19 loss and grieving experienced by LGBTQIA+ individuals, including lack of work and livelihood, social isolation, loss of community connection, lack of physical and mental health support, and lack of identity/self‐affirmation [25]. Although impactful measures to address long COVID are available to some extent, there is a lack of efficient and compelling vision in marginalized populations (i.e., color and LGBTQIA+ individuals) and their subgroups (e.g., the lesbian, gay, bisexual, transgender, queer, questioning, intersex populations, as well as low‐income groups) as a language like a Systems Thinking model to conceptualize such complicated challenges with a focus on the smell loss. This has not yet explored how loss of smell affects resilience, personal growth, and the reconstruction of meaning among LGBTQIA+ individuals with long‐term COVID‐19. This understanding could eventually translate research findings into effective health practices for marginalized communities.

Hence, the primary goals of the article are (1) to study the impact of long COVID on the marginalized population, in particular, the vulnerable LGBTQIA+ communities, and to visualize how sensory dysfunction can impact physical health, mental health, financial stability, meeting basic needs, and feelings of social connectedness (2) using a preliminary causal loop diagram (CLD), Systems Dynamic, and model‐based systems thinking (MBST) to conceptualize and design advanced, robust, and future sensitive research trials. Our findings highlight the urgent need for further research into long COVID and the creation of specific interventions and treatments to lessen its effects on marginalized communities using Systems Thinking. This could also broadly benefit individuals experiencing anosmia.

## 2 Methods

Advanced search options were utilized on electronic databases such as PubMed, Scopus, and Science Direct to identify articles using terms like “olfactory dysfunction,” “anosmia,” “smell,” “olfaction,” “neurological manifestations,” “neuropsychiatric disorders,” “cognitive decline,” “treatment,” “olfactory training,” “smell training,” “COVID‐19,” “SARS‐CoV‐2,” “coronavirus disease,” “Systems Thinking,” “Systems Dynamic,” “causal loop diagram,” “LGBQTA+,” “long COVID,” “natural products,” and “neurocognitive disorders” in various combinations. The suitability of articles for inclusion in this review was assessed on the basis of their relevance to COVID‐19‐related anosmia and neurological symptoms, their exploration of olfactory dysfunction with neurocognitive disorders, and their discussion of treatments, particularly olfactory training interventions for anosmia. Both human and animal studies were considered, and there were no time constraints on the publication date. Articles were excluded if they had misleading titles, unclear methodologies, or weak study designs. The reference lists of selected articles were also reviewed for pertinent information.

## 3 Results

The search across electronic databases returned ∼2000 articles. These articles were then evaluated for their relevance, and their bibliographies were checked for additional relevant studies. Ultimately, 100 articles met the criteria and were included in this review by consensus among all authors. Most studies came from various global regions and encompassed human and animal research.

### 3.1 COVID‐19 Infection and Its Role on Olfactory Dysfunction

**FIGURE 1:**
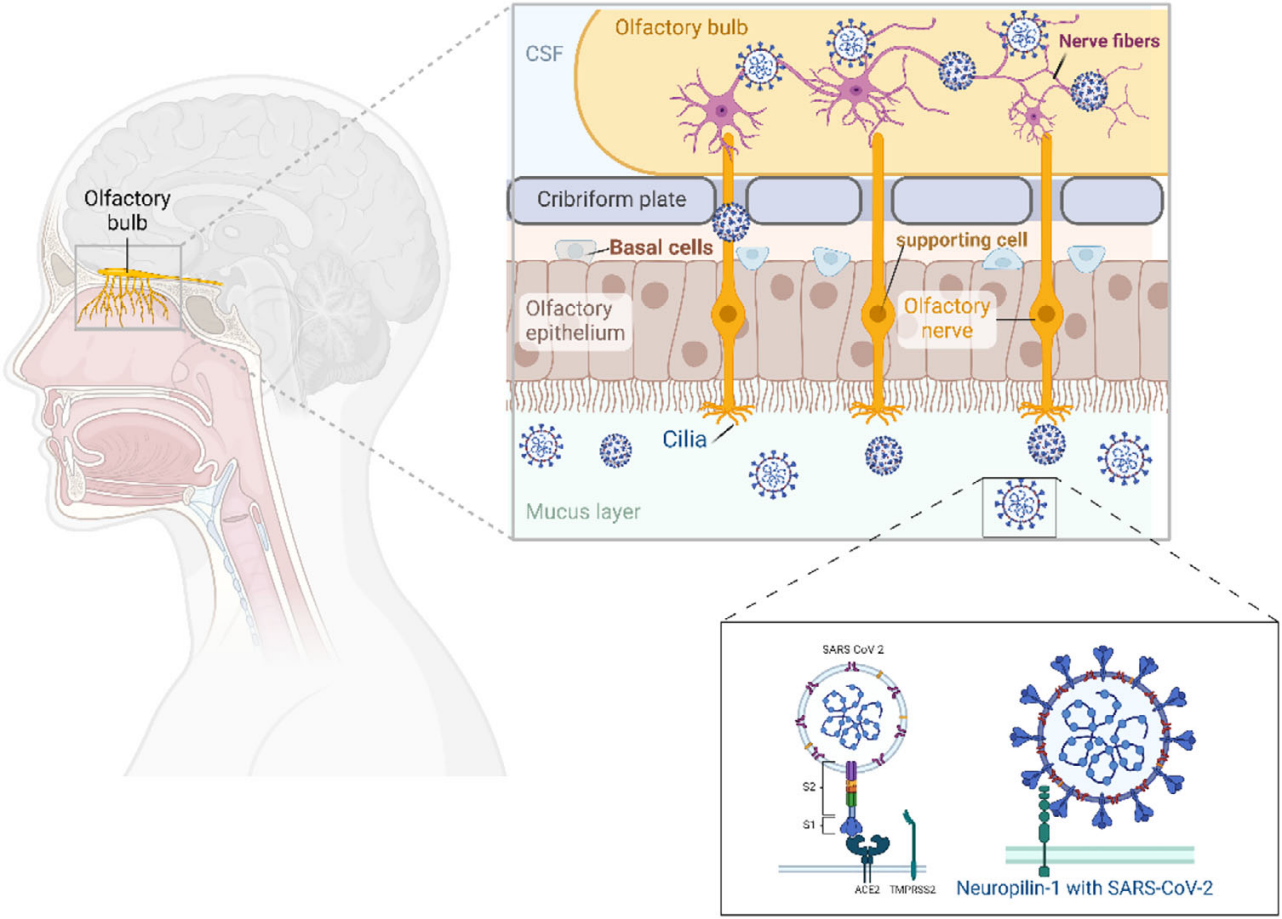
Illustrates the mechanisms by which SARS‐CoV‐2 reaches the central nervous system and causes olfactory dysfunction in COVID‐19 and how the virus enters through the olfactory pathway, initially affecting the olfactory epithelium and then progressing to the olfactory bulb via axonal transmission. The SARS‐CoV‐2 can also disseminate through the systemic circulation. This may lead to the infection of the endothelial cells and damage to the blood–brain barrier (BBB) or enable infected immune cells to infiltrate the brain, causing hematogenous spread. ACE‐2, angiotensin‐converting enzyme 2; COVID‐19, coronavirus disease‐2019; OSN, olfactory sensory neuron; SARS‐CoV‐2, severe acute respiratory syndrome coronavirus 2; TMPRSS2, transmembrane serine protease 2. *Source:* This figure was created/adopted with BioRender.com.

Olfactory impairment, one of the more unique symptoms of COVID‐19 infection, can persist beyond recovery, and therefore, olfactory training interventions have seemed to be of considerable current interest [13, 18, 20, 26, 27]. Olfactory dysfunction results from viral damage to the olfactory epithelium, which blocks the ability of scents to bind with olfactory receptors (Figure 1) [13, 28, 29]. It is believed that SARS‐CoV‐2 causes anosmia by targeting the olfactory epithelium. This tissue, lining the olfactory cleft in the nasal cavity, contains the primary sensory neurons that detect odors, a layer of sustentacular cells, and basal stem or progenitor cells that continually renew the epithelium [30, 31]. When the inflammatory molecules (e.g., cytokines) are released, they can penetrate the blood–brain barrier, impacting the olfactory system and causing inflammation and damage to olfactory neurons [30]. The degree to which olfactory neurons can regenerate after damage remains unclear [32]. Although some research indicates that olfactory neurons can regenerate after injury, other studies suggest that this regenerative ability diminishes with age. Datta et al. showed that in most instances, SARS‐CoV‐2 infection is unlikely to cause permanent damage to the olfactory neural circuits or lead to enduring anosmia [33, 34]. As presented in Figure 1, the presence of angiotensin‐converting enzyme 2 (ACE2) and the pro‐protein convertase furin (PCF) on brain microvascular endothelial cell membranes facilitates SARS‐CoV‐2 infection. Although endothelial cells express ACE2 along with TMPRSS2, cathepsins, and other SARS‐CoV‐2 entry factors, the levels of ACE2 and TMPRSS2 are lower compared to those in nasal epithelium and pulmonary alveolar Type 2 cells. SARS‐CoV‐2's spike protein has a furin cleavage site, which is not present in SARS‐CoV. Research by Cantuti‐Castelvetri et al. demonstrated that neuropilin‐1 (NRP1), which binds to furin‐cleaved substrates, enhances SARS‐CoV‐2 infectivity [35]. NRP1 is highly expressed in respiratory and olfactory epithelium, particularly in endothelial and epithelial cells. Daly et al. discovered that the furin‐cleaved S1 fragment of the spike protein binds directly to cell surface NRP1 and that blocking this interaction with a small‐molecule inhibitor or monoclonal antibodies reduces viral infection in cell cultures [35]. Exploring the role of NRP1 in SARS‐CoV‐2 infection could identify potential targets for antiviral treatments in the future. It is important to note that post‐viral olfactory dysfunction is not exclusive to SARS‐CoV‐2 but can also result from other respiratory infections [20, 21, 36].

Researchers actively seek effective therapies for individuals experiencing anosmia or diminished sense of smell following an infection [12, 28, 37–39]. Studies have shown positive results in structured olfactory training for individuals to regain their sense of smell and taste after COVID‐19 [6, 20, 21, 40, 41]. Moreover, natural products containing certain nutrients and metabolites have shown promise in helping patients with taste and smell loss and overall nutrition [38, 42–48]. However, attempts at fortifying flavors and using zinc and antioxidant products have not been scientifically supported as effective treatments [49]. The altered brain activity in areas that process olfactory information suggests a direct neurological impact that may be amenable to interventions like olfactory training [39, 41, 50]. Hence, further research is needed to understand the effects of an individual's diet on their taste and smell dysfunction, emotional distress, and isolation after recovering from COVID‐19. Of course, society must address long‐lasting nasal inflammation in a new light to thrive post‐pandemic.

### 3.2 Systems Thinking

Systems Thinking involves a broader understanding of a system and improving its overall quality, including its various elements and group interactions. When navigating Systems Thinking intervention, there are five crucial steps to follow. These include root cause analysis, selecting and focusing on strong‐leverage areas (where system interventions can significantly impact), systems design, measures to counteract unintended consequences, and continuous learning from the entire process. These steps are not necessarily mandatory, as the approach may vary depending on the problem being addressed. The first step involves identifying the problem, setting intervention goals, and gathering information about the situation. In Systems Thinking, a leverage point refers to a spot where a solution element can be applied to a system. Low leverage points mean that small changes will result in small behavioral changes, whereas high leverage points mean that small changes will lead to significant behavioral changes [51, 52, 53, 54, 55, 56].

Systems Thinking helps (1) calibrate assumptions to information and quantify them to organize explicit modeling and crucial for research studies and professional interventions, (2) study how system elements interact and construct the desirable system (e.g., in public health) using modeling tools such as CLD, (3) identify the primary causes of system defects with feedback‐loop structures and ultimately, and (4) develop interventions that are appropriate to the situation.

However, this practice requires significant research, evaluation, and evidence‐based decision‐making [51, 53, 55–59]. Using CLDs involves selecting critical variables and leverage points, investigating their relationships and behavior over time, and generating intervention strategies [60]. Dynamic modeling and testing are then conducted to refine the models, including verifying model equations, selected parameters, and behavior over time [61, 62, 63]. Scenario planning and modeling are then employed to evaluate the effectiveness of the devised strategy in the face of change and uncertainty. Finally, these refined models can be deployed in microworlds to test hypotheses and facilitate continued learning [60, 61].

### 3.3 Real‐World Applications of Systems Thinking in the Pandemics

**FIGURE 2:**
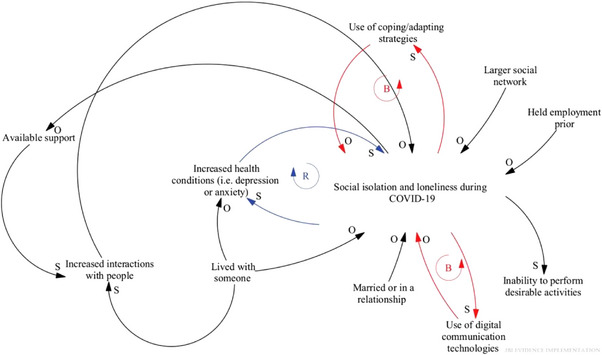
The impact of social isolation and loneliness during the COVID‐19 pandemic. This depicts the impact of social isolation and loneliness during the pandemic. The directional arrows between the “O” and “S” designations indicate the indirect and direct effects of the variables. *Source:* Reproduced with copyright permission [1].

Long COVID clinics are adopting a holistic approach to treatment, recognizing the multifaceted symptoms affecting patients. This approach aligns with the Systems Thinking model, advocating for integrated care that comprehensively addresses mental, physical, and social health needs. Real‐world applications of this model can significantly benefit from recognizing the specific vulnerabilities and needs of LGBTQIA+ individuals, such as those related to gender‐affirming treatments and mental health services [64]. For instance, Figure 2 highlights where the variables can move in the same direction or indirectly when affected [56]. As clarified in the diagram, the effects of social isolation and loneliness during the COVID‐19 pandemic have direct and indirect impacts, with directional arrows between the “O” and “S” markers highlighting the relationships among the variables [56]. Having a more comprehensive social network, good communication experiences, meaningful, and effective interactions with people, being employed before COVID‐19, being in a relationship or marriage, and living with someone are all factors that decrease social isolation and loneliness in older adults. However, as these public health risks increase, anxiety, depression, and suicide rates are also expected to rise. Balancing loops and digital tools used for communication can promote progress in coping and adaptation to mitigate social isolation and loneliness [56, 65].

**FIGURE 3:**
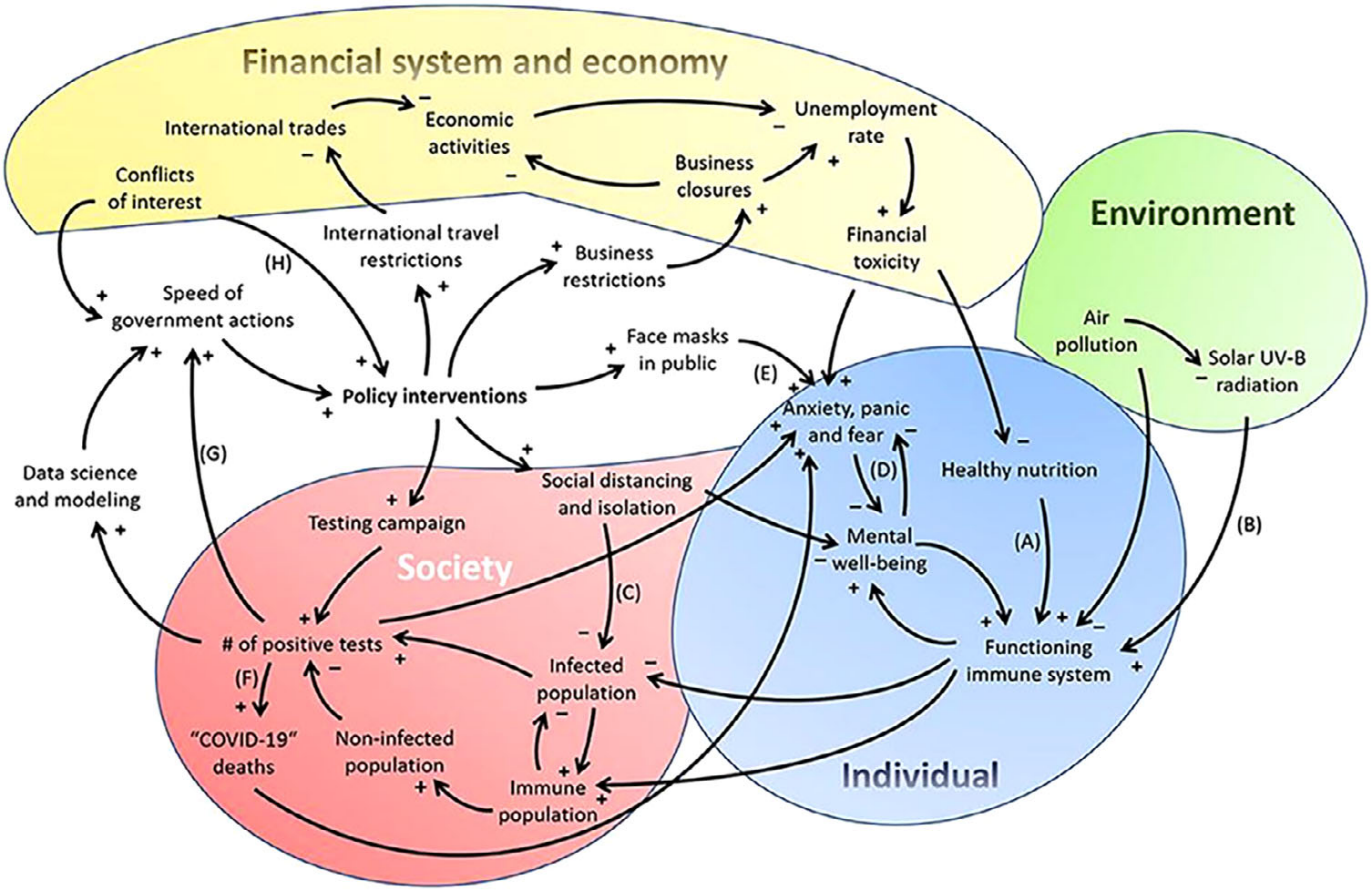
A preliminary CLD that depicts complex‐related SARS‐CoV‐2 crisis’ environmental–individual–socio–economic–political system. *Source:* Reproduced with copyright permission [2].

Using adjustable parameters, the CLD model identifies leverage points to reduce social isolation and loneliness during the COVID‐19 pandemic [60]. To provide comfort and care to those in need during the end‐of‐life period and career, education and resources that foster healthy adaptive coping approaches would be beneficial. Additionally, the CLD strategy could be employed to identify divergent healthcare‐related factors that lead to medication errors, diagnostic failures, and increased readmission rates. The interrelationships among various parameters across a CLD and their contributions to a critical issue can be monitored through numerous interviews and group conversations, providing information to clinicians and health service employers [56]. Sahin et al. developed a preliminary CLD that incorporated information about the pandemic's impact on the economy, environment, individuals, and society [66]. The diagram showed how these elements were interrelated during the COVID‐19 outbreak, assessed government activities in response to the pandemic [31, 66], and outlined numerous feedback loops related to the SARS‐CoV‐2 issue within the environmental system. Although this CLD provides a helpful foundation for shaping policy interventions aimed at SARS‐CoV‐2 and other future infectious disease outbreaks, it overlooks the individual‐level systems central to various medical disciplines. Thus, the adapted CLD on the basis of Sahin et al.’s model was developed by Klement (Figure 3) [59].

Ben Hassen et al. have used a susceptible–infectious–removed (SIR)‐Poisson model to predict the range of infections [67]. At the same time, Hoch et al. developed the SIR‐susceptible (SIRS) model to analyze the transmission of infectious diseases in a multigroup system. Zhao et al. have investigated the transmission dynamics of SARS‐CoV‐2 to develop individual case management strategies and reduce the number of super‐spreaders [68]. Rahimi et al. in‐depth studies promoted peaceful and inclusive countries for sustainable development, initiating the bidirectional link between COVID‐19 and the environment [69].

### 3.4 Challenges in Studying LGBTQIA+ Populations in the Face of COVID‐19 Pandemic

Studying the LGBTQIA+ populations during the COVID‐19 pandemic presents unique challenges. As researchers and policymakers strive to understand and mitigate the effects of the pandemic, the LGBTQIA+ community faces distinct health disparities that general studies or interventions may not adequately address. These challenges are compounded by historical and ongoing stigmatization, as well as a lack of comprehensive data that is sensitive to the specific needs and experiences of LGBTQIA+ individuals. Consequently, tailored approaches are essential to ensure that the health policies and research strategies implemented are genuinely inclusive and effective in supporting this vulnerable population during such a global health crisis. Providing sufficient mental healthcare for the LGBTQIA+ community is essential to bridge existing disparities. However, there are scarce opportunities and resources available for mental health professionals who specialize in LGBTQIA+ affirmative care. Fish et al. recently implemented a virtual training program called the Sexual and Gender Diversity Learning Community (SGDLC) to help mental health professionals become more culturally competent when working with LGBTQIA+ communities during the COVID‐19 pandemic [70]. Their findings suggest that the virtual SGDLC training program was successful due to its feasibility and satisfaction, as guided by the expanded RE‐AIM model [70]. However, concerns about scaling up this education beyond a viral reality remain unresolved. Lucas et al. highlighted how LGBTQ+ individuals have suffered during the HIV/AIDS pandemic of the 1980s–1990s and the current COVID‐19 pandemic, leading to loss and grief experiences that have often gone unacknowledged [25].

Furthermore, research studies indicated that LGBTQIA+ individuals face significant disparities in healthcare access and outcomes, which have been exacerbated during the COVID‐19 pandemic [22, 56, 70, 71]. For example, studies show that LGBTQ adults report higher rates of job loss and mental health struggles compared to their non‐LGBTQ counterparts due to the pandemic. This context is crucial for applying MBST approaches to address the physical symptoms of long COVID and the psychological stresses that may be more pronounced in this community [24]. Another example is a notable lack of systematic monitoring for LGBTQIA+ health in the United Kingdom, a gap highlighted by the UK government's 2018 LGBT action plan, which aimed to prioritize LGBTQIA+ needs within the National Health Service (NHS) [72, 73].

The pandemic has also intensified other challenges for LGBTQIA+ individuals, including an increase in domestic abuse and significant barriers to accessing healthcare. During the lockdown, nearly 40% of LGBTQIA+ people reported missing medical appointments, with delays in medical procedures and restricted access to essential treatments disproportionately affecting trans individuals [74]. Despite these challenges, the pandemic has galvanized the LGBTQIA+ voluntary sector to enhance service offerings and support, demonstrating resilience despite financial constraints. Emphasizing the importance of community integration, Brady advocates incorporating LGBTQIA+‐specific services and community feedback into mainstream healthcare practices to foster inclusivity and improve care delivery effectively [25, 75–77]. Overall, the COVID‐19 pandemic has further exposed and deepened these health disparities, underscoring the urgent need for improved healthcare approaches and data collection for the LGBTQIA+ community [77]. Phillips emphasized the need for systematic monitoring of health data concerning gender identity and sexual orientation to combat disparities [73]. A qualitative evidence synthesis conducted by Lucas et al. identified several categories of COVID‐19 loss and grieving experienced by LGBTQIA+ individuals, including lack of work and livelihood, social isolation, loss of community connection, lack of physical and mental health support, and lack of identity/self‐affirmation [25].

Currently, there is no routine national monitoring of sexual orientation or gender identity, which significantly impedes a thorough understanding of how the pandemic impacts LGBTQIA+ individuals [78]. Available data already show substantial health inequalities, such as elevated rates of disabilities and chronic conditions among trans and nonbinary people and higher obesity prevalence among lesbian and bisexual women [79, 80]. The mental health of LGBTQIA+ individuals also suffers due to these systemic failures, with this group starting from a lower baseline than the general population. Conditions such as depression, self‐harm, and suicidality are alarmingly common, particularly among minority ethnic and transgender or nonbinary people, and have worsened due to pandemic‐induced isolation and diminished support from chosen families and community networks [24].

Over half of gender minority people and one‐third of cisgender sexual minority people reported worsening physical health. These findings are similar to data by Heslin and Hall [81], who found that sexual minority people have higher prevalences of many underlying conditions associated with severe COVID‐related illnesses than heterosexual people. These increased risks for the LGBTQIA+ population likely stem from reports of stigmatizing experiences in healthcare and lack of provider knowledge [82]. Another important consideration is the close living proximity of dense marginalized communities, which can increase the risk for infections [83] and subsequent poorer physical health [84].

### 3.5 Long‐Term Smell Loss Experiences After COVID‐19 in LGBTQTA+ Families

The sense of smell relies on specialized olfactory receptor neurons in the olfactory epithelium of the nasal cavity [19]. Odorant molecules bind to receptors on the surface of these neurons, leading to the generation of a nerve impulse, which is relayed to the olfactory cortex; it is here that smells are perceived [85]. Changes to the sense of smell and taste can deprive a patient of the pleasure of eating, which can have significant psychological, social, and physiological effects [16]. Anosmia has been associated with food aversion, reduced social interactions, anxiety, depression, malnutrition, and anorexia [2, 76, 86]. Most patients will regain their sense of smell after a few weeks, but around 5% of people infected with COVID‐19 still report smell and taste dysfunction 6 months later [87, 88, 89, 90].

Smell loss can contribute to feelings of anxiety and depression, which are already prevalent in the LGBTQIA+ community due to social stigma and discrimination [24, 25, 72, 73, 74, 82, 84]. This sensory loss may exacerbate existing mental health challenges, leading to increased stress within family dynamics [78, 80, 82]. Smell is crucial in social bonding, from sharing meals to intimate personal interactions. Loss of this sense can alter these experiences, potentially leading to social withdrawal or isolation. For LGBTQIA+ families, who may already face challenges in social acceptance, this can exacerbate feelings of alienation or loneliness [24, 73, 77, 84]. The stress and anxiety associated with long COVID, including symptoms like smell loss, can have significant mental health implications [14, 65, 76]. Research indicates that LGBTQIA+ individuals generally report higher levels of stress and mental health issues compared to their heterosexual counterparts. The added burden of long COVID symptoms can intensify these issues, making mental health support crucial for affected LGB individuals and their families. Ongoing health issues associated with long COVID, such as chronic fatigue and smell loss, can complicate this population's daily routines and family dynamics [2–4, 64, 65, 91, 92]. This can affect caregiving roles within families, alter relationship dynamics, and increase the need for medical and supportive care, which may not always be readily accessible or affirming for LGBTQIA+ individuals [24, 74, 76].

The economic repercussions of long‐term COVID can also be significant; specifically, the LGBTQIA+ individuals who often face economic disparities and employment discrimination, and long‐term COVID may lead to job loss or reduced working hours, further straining family resources [75]. This can affect their sensory perception, cognitive functions, and emotional well‐being [82]. Smell loss can profoundly affect social interactions, often centered around meals and personal interactions involving scent. For individuals experiencing smell loss, these activities can become less enjoyable, leading to increased social isolation. For the LGBTQIA+ communities, which already face higher risks of isolation, this can exacerbate feelings of loneliness and disconnection. The stress from ongoing health concerns, compounded by potential job loss and economic insecurity within the LGBTQIA+ communities, underscores the need for targeted mental health support during and following the pandemic [3, 25, 70, 73, 77, 84].

#### 3.5.1 Interconnected Aspects of Long COVID in LGBTQIA+ Patients With a Focus on Olfactory Dysfunction

**FIGURE 4:**
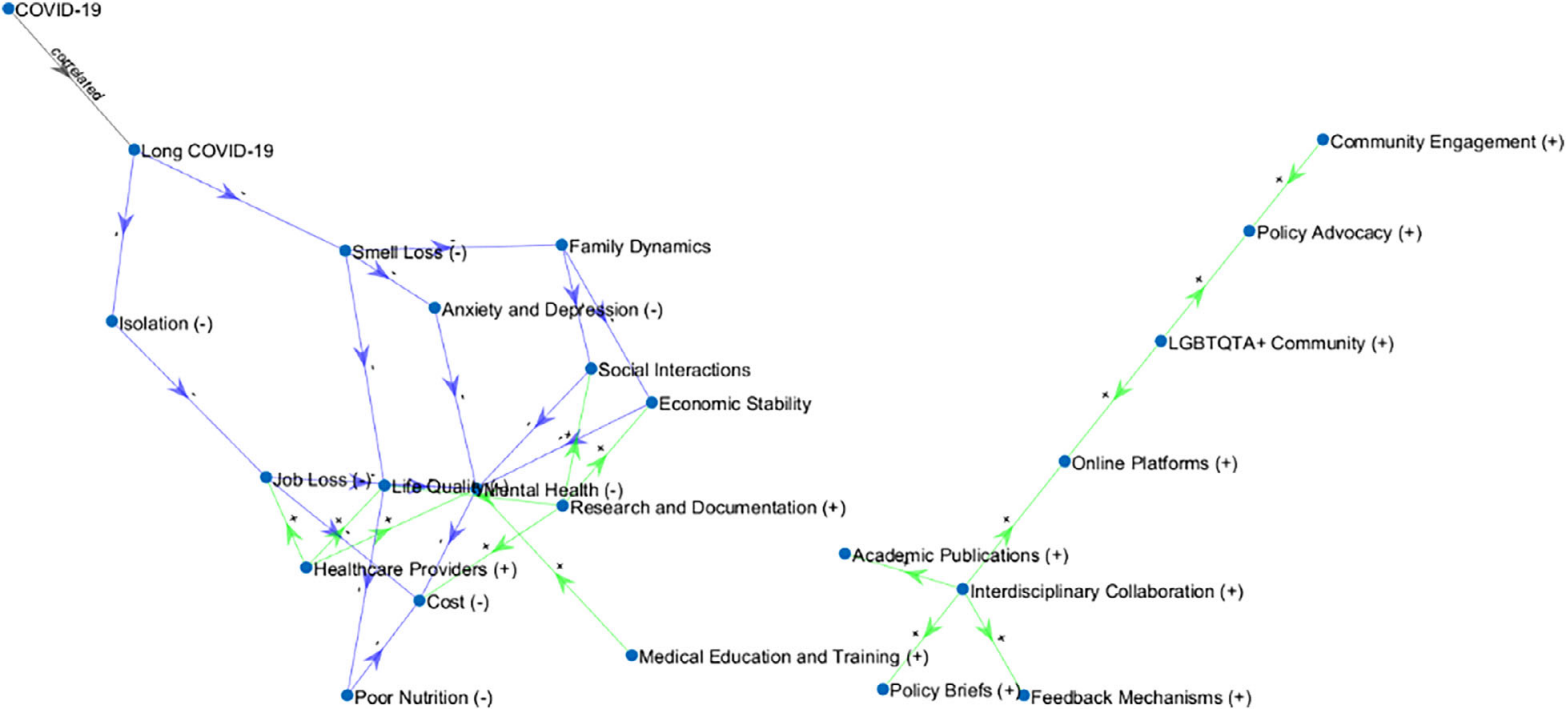
The System Dynamic model displays the key variables and their relationships, highlighting the complex interdependencies and feedback loops in the system. The nodes represent the different factors involved, whereas the arrows indicate the direction and nature of influence among these factors. *Nodes:* Added all the nodes including those for positive and negative interactions. *Edges:* Defined all the edges to match the nodes correctly and added interaction types (+) for positive, (−) for negative, and “correlated” for correlation). *Edge labels:* Added edge labels to display (+) above the arrows and (−) below the arrows. *Edge colors:* Set the color of the edges based on the interaction type: green for positive interactions, blue for negative interactions, and black for correlated interactions.

As discussed, sudden smell loss is one of the early symptoms of COVID‐19. Although it is stated that the loss of smell and taste following COVID‐19 improves within a few weeks, some cases do not improve for a long time. Our MBST model (Figure 4) offers fresh insights into the effects of long‐term COVID and the interconnected aspects of olfactory loss in patients from diverse communities, including LGBTQIA+, and how smell loss may complicate the Systems Thinking‐based models and make long‐term COVID worse in this specific population.

This study aims to reveal long‐term smell loss experiences after COVID‐19. Nutrition and appetite, personal hygiene, threats to safety, and emotional changes were the main themes created by the authors and were the areas where participant expressions focused. The participants used oral/nasal corticosteroid therapy for smell loss and received short‐term olfactory training but could not find a solution. Long‐term smell loss problems, neglected during the pandemic, should be carefully evaluated due to their harmful effects. Understanding and focusing on the adverse effects of loss of smell may contribute to the solution of long‐term smell loss problems. A detailed outline to operationalize the approach is as follows:

***Key variables***

1. **Mental health**
  - Represents the psychological well‐being of individuals, affected by long COVID.
  - Includes factors like anxiety, depression, and cognitive impairments.
2. **Life quality**
  - Represents the overall well‐being and daily functioning of individuals.
  - Includes factors like physical health, ability to perform daily activities, and social interactions.
3. **Economic stability**
  - Represents the financial status and employment situation of individuals.
  - Includes factors like job loss, reduced working hours, and healthcare costs.

***System boundaries***

- **Population:** Focus on individuals affected by long‐term COVID‐19, specifically emphasizing marginalized communities such as LGBTQ and low‐income groups.
- **Geographical scope:** Depending on data availability, this could be national, regional, or community‐specific.
- **Time horizon:** To capture long‐term effects, the model should cover the period from the onset of COVID‐19 to several years after the pandemic.

***Feedback loops***

1. **Reinforcing loop (positive feedback)**
  - **Mental health **→** economic stability **→ **mental health:**
    ▪ Poor mental health can lead to job loss or reduced productivity, worsening economic stability.
    ▪ Economic instability can further deteriorate mental health, creating a reinforcing loop of decline.
2. **Balancing loop (negative feedback)**
  - **Interventions **→** mental health **→ **economic stability:**
    ▪ Effective interventions (e.g., medical education and community engagement) can improve mental health.
    ▪ Improved mental health can enhance economic stability by increasing productivity and job retention, which can, in turn, support further interventions.
3. **Balancing loop (negative feedback)**
  - **Life quality **→ **mental health **→ **life quality:**
    ▪ Poor life quality due to long COVID symptoms can lead to mental health issues.
    ▪ Addressing mental health can improve life quality, breaking the cycle of decline.

***Planned analyses***

1. **Sensitivity analysis**
  - **Purpose:** To understand how changes in key parameters affect the model's outputs.
  - **Method:** Vary parameters such as the rate of job loss, effectiveness of interventions, and healthcare costs to see their impact on mental health and economic stability.

***Operationalizing the approach***

1. **Data collection and model calibration**
  - **Data sources:** Use data from existing studies on long‐term COVID impacts, mental health, and socioeconomic conditions.
  - **Calibration:** Adjust model parameters to ensure the outputs match observed data.
2. **Model simulation and validation**
  - **Simulation:** Run the model under different scenarios to predict future states and identify potential intervention points.
  - **Validation:** Compare model outputs with real‐world data and expert opinions to validate the accuracy and reliability of the model.

#### 3.5.2 Modeling and Simulation of Systems Thinking Using MATLAB

We also provided a MATLAB script to visualize the interactions and impacts in Systems Thinking correctly. For instance, the scenario is assessing the effects of increased mental health support on the LGBTQIA+ community affected by long COVID:

- **Simulation:** Increase the availability of mental health services and measure the resulting changes in mental health, life quality, and economic stability.
- **Outcome:** Identify the most effective interventions and provide recommendations for policymakers to support the LGBTQ community.

#### 3.5.3 Enhancing Healthcare Outcomes With MBST: Advantages for Providers and the LGBTQIA+ Community

Our MBST can become a practical and influential tool in enhancing healthcare provision and advocacy for the LGBTQIA+ community, ultimately leading to better health outcomes and reduced disparities. Overall, the MBST model can facilitate successful implementation in real‐world clinical settings, providing valuable insights into the subtleties and dynamics of olfactory deficiencies and devising appropriate interventions that consider all communities, including the LGBTQIA+.

##### 3.5.3.1 Education and Training

It has offered targeted training sessions on Systems Thinking methodologies for healthcare providers, including case studies specific to LGBTQ health issues. These sessions demonstrate how Systems Thinking can uncover complex interdependencies and lead to more effective interventions. Workshops could be integrated into medical education and ongoing professional development programs [93].

##### 3.5.3.2 Community Engagement

It has involved the LGBTQIA+ community in developing and applying Systems Thinking models. This could be done through community forums, workshops, and participatory research projects where community members collaborate with healthcare providers to map the systems affecting their health outcomes. Engaging the community in this way ensures that the models reflect real‐world experiences and needs.

##### 3.5.3.3 Visual Tools and Resources

It has developed intuitive visual tools that simplify systems thinking concepts. Diagrams, interactive models, and digital platforms can help providers and community members visualize the complex systems at play. These tools should be designed with user‐friendly interfaces to accommodate individuals without a background in Systems Thinking.

##### 3.5.3.4 Policy Advocacy

It has used insights derived from Systems Thinking to advocate for policies that address the systemic barriers faced by LGBTQIA+ individuals. Healthcare providers, backed by robust systems analysis, can play a pivotal role in policy discussions, advocating for changes that improve health access and outcomes for marginalized communities.

##### 3.5.3.5 Research and Documentation

Making this information/diagram widely available through academic publications, policy briefs, and online platforms can serve as a blueprint for other healthcare providers and communities.

##### 3.5.3.6 Interdisciplinary Collaboration

It can foster collaborations between healthcare providers, systems thinkers, social scientists, and LGBTQIA+ advocacy groups. These multidisciplinary teams can create more holistic and comprehensive models that factor in medical, social, psychological, and economic elements.

##### 3.5.3.7 Feedback Mechanisms

It has established continuous feedback loops that allow healthcare providers and community members to update and refine systems models on the basis of new information and outcomes. This adaptability is crucial in Systems Thinking and ensures that the models remain relevant and practical.

#### 3.5.4 Systems Dynamic Model

**FIGURE 5:**
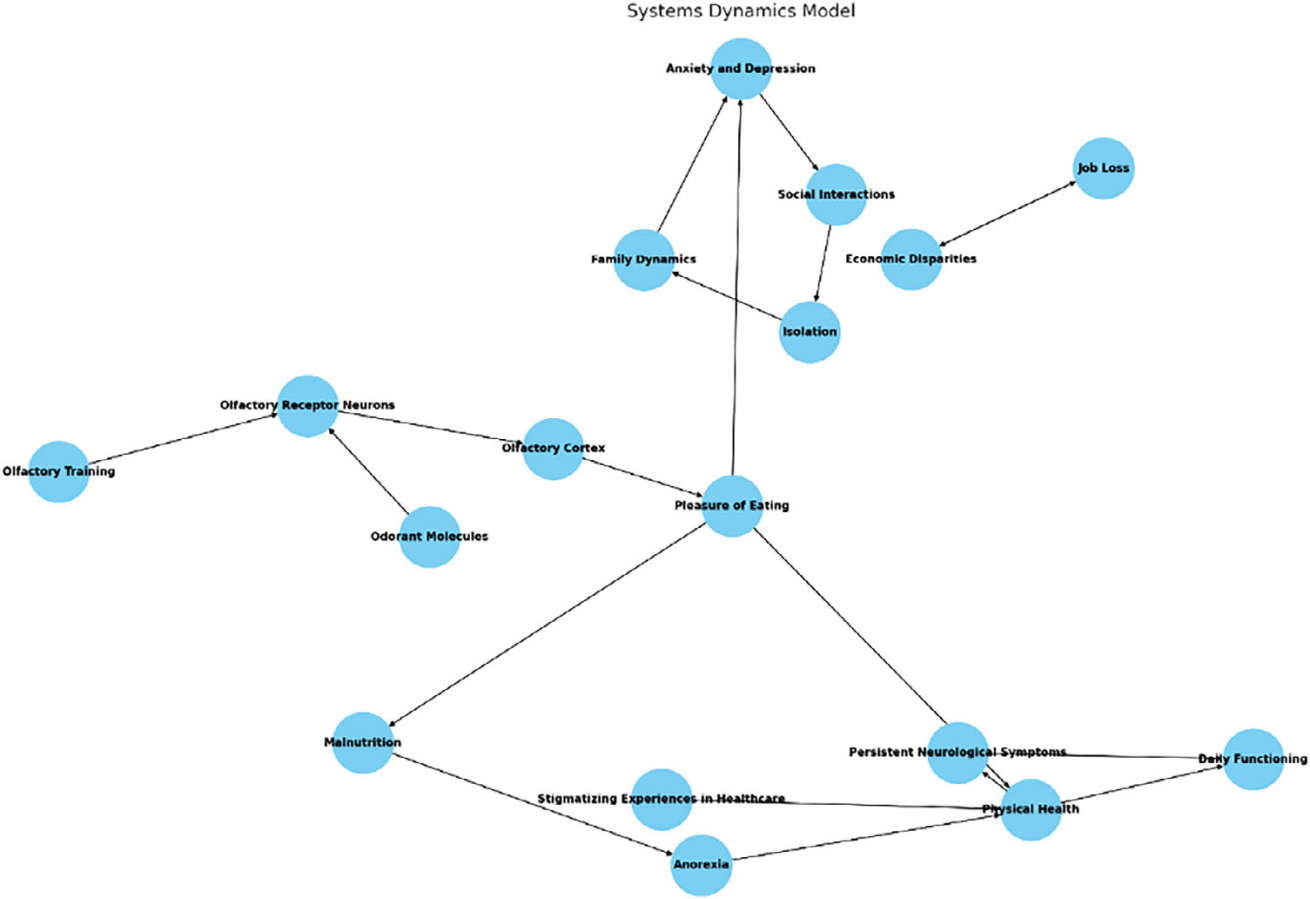
System Dynamics model: (A) odorant molecules bind to olfactory receptor neurons, which send signals to the Olfactory Cortex, leading to the perception of smell; (B) the perception of smell influences the pleasure of eating, which can affect anxiety and depression levels; (C) anxiety and depression impact social interactions, potentially leading to isolation and changes in family dynamics, family dynamics feedback into anxiety and depression, creating a loop; (D) pleasure of eating also affects malnutrition, which can lead to anorexia and deteriorated physical health; (E) physical health influences persistent neurological symptoms and overall daily functioning; (F) stigmatizing experiences in healthcare impact physical health, exacerbating conditions; (G) economic disparities and job loss form a feedback loop, affecting each other; and (H) olfactory training can help improve olfactory receptor neurons, potentially restoring the sense of smell and breaking negative cycles.

The Systems Dynamic diagram displays the key variables and their relationships, highlighting the system's complex interdependencies and feedback loops. The nodes represent the factors involved, whereas the arrows indicate the direction and nature of influence among these factors. This visual representation helps understand how various factors interact and influence each other within the system (Figure 5).

##### 3.5.4.1 Causal Loop Diagram

**FIGURE 6:**
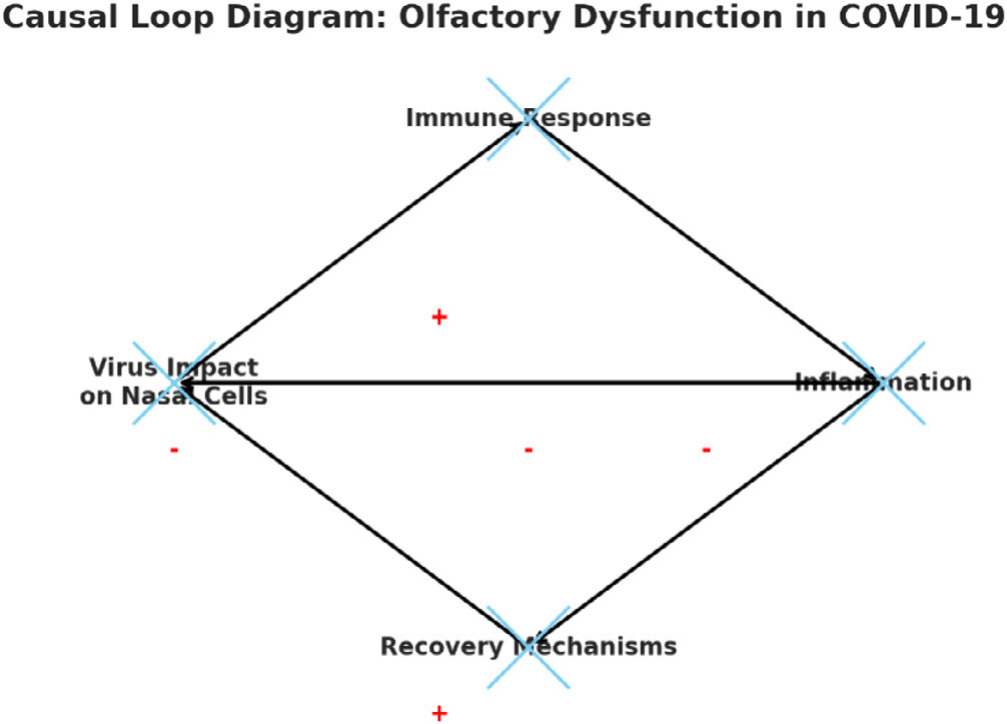
Proposed causal loop diagram: olfactory dysfunction in COVID‐19. The arrows indicate the direction of influence between these components, with positive (+) and negative (−) feedback loops marked in red to show how these interactions can either exacerbate or mitigate the effects of olfactory dysfunction.

The CLD in Figure 6 illustrates the interactions among the main components involved in olfactory dysfunction caused by COVID‐19:

- **Virus impact on nasal cells:** The starting point is when the virus causes damage or changes to nasal cells, which is crucial for smell detection and leads to olfactory dysfunction.
- **Immune response:** The body's defense mechanism against the virus, including inflammation and releasing immune cells and cytokines.
- **Inflammation:** Part of the immune response that affects explicitly the nasal area and further impacts olfactory function.
- **Recovery mechanisms:** The body's efforts to repair and return to normal function, including regenerating nasal cells and reducing inflammation.

#### 3.5.5 Limitation of Study

As the current Systems Thinking model is designed without qualitative analysis, the model may fail to capture the nuanced experiences, emotions, and personal narratives of LGBTQIA+ individuals. However, these narratives are crucial for understanding profoundly personal and sociocultural aspects of health and well‐being to design this sensitive research study, accounting for the diversity within the LGBTQIA+ community, including socioeconomic status, race, age, and gender identity. Qualitative research provides insights into how these factors influence individual experiences with health, discrimination, and access to services.

Although Systems Thinking is excellent for identifying major feedback loops and interactions, it may not capture subtler dynamics that qualitative insights can reveal. These might include internalized stigma, fear of disclosure in healthcare settings, or subtle forms of social support that significantly impact health outcomes. In addition, qualitative research helps ensure that studies are ethically grounded in the lived realities of participants, promoting respect and accuracy in research findings.

Natural products, including diarylheptanoids, terpenoids, and flavonoids (e.g., kaempferol and liquiritigenin), have shown potential in olfactory dysfunction within and after recovery from coronavirus infection by synergistically inhibiting the SARS‐CoV‐2 MPRO and PLPRO in vitro. Natural flavonoids (e.g., quercetin and luteolin) impede the entry of the virus into host cells, interfere with neuroinflammation, and decrease cognitive decline [42, 94–96]. However, their effects and efficacies are still under evaluation in clinical trials. Numerous therapies have been proposed and explored. What has consistently emerged as most effective, both clinically and in studies, is a technique known as olfactory retraining.

**FIGURE 7:**
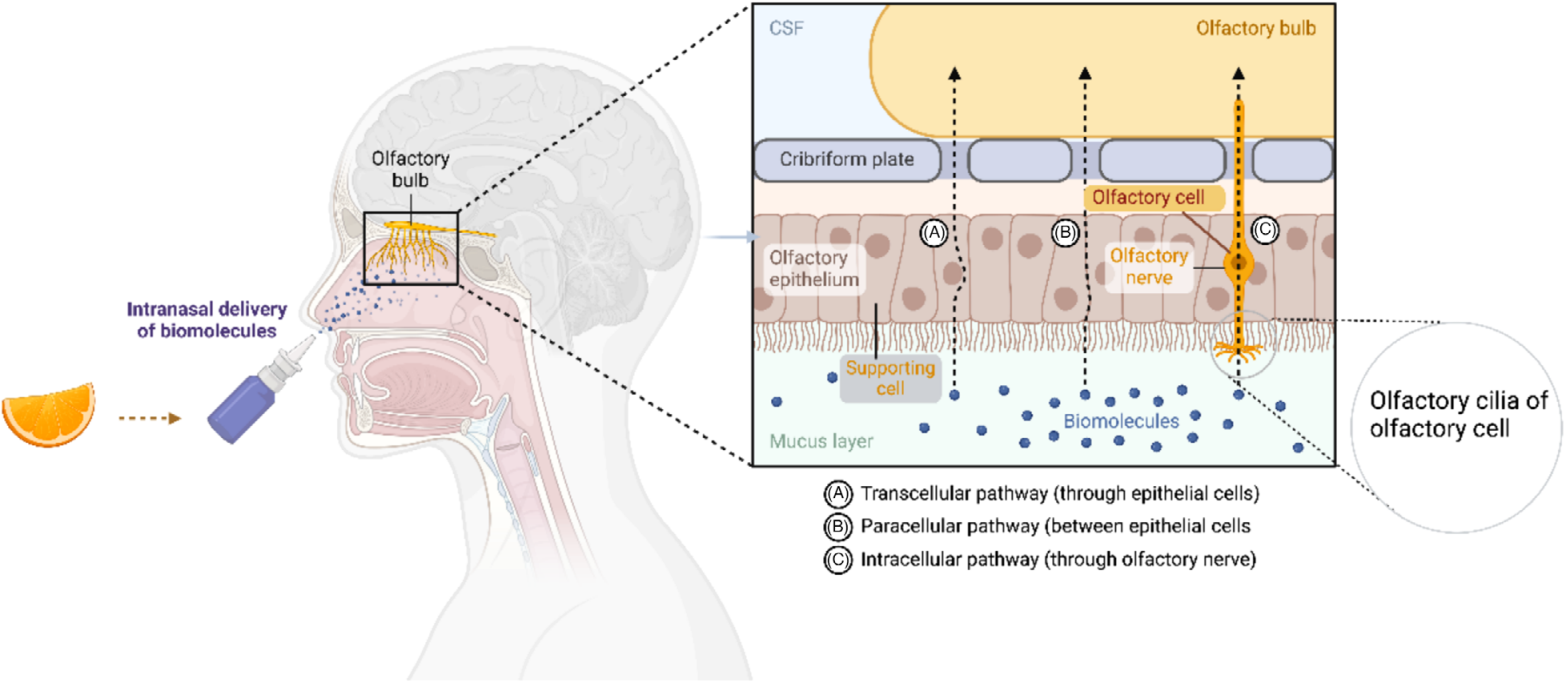
The image illustrates the pathway of olfactory transmission and potential sites for therapeutic intervention in the context of smell loss, particularly relevant in conditions such as COVID‐19‐induced anosmia. The diagram is split into two major parts: (A) overall pathway: Shows the pathway from the nostrils to the brain, highlighting how biomolecules, possibly therapeutic agents, are delivered intranasally and move toward the olfactory bulb; (B) detailed mechanism: (1) the olfactory epithelium and its components like olfactory cells and supporting cells, (2) the passage of scent molecules binding to receptors on the olfactory cells, and (3) the transmission of signals via the olfactory nerve to the olfactory bulb and then to the brain for scent perception. (C) Intracellular pathway (through olfactory nerve) *Source:* This figure was created/adopted with BioRender.com.

The nerves responsible for taste and smell can heal and regenerate, a process known as neuroplasticity. Olfactory Training uses distinct scents to retrain the olfactory system (Figure 7). It is a safe, self‐driven option that utilizes primary odors like rose, lemon, cloves, or eucalyptus to stimulate the olfactory nerves and help regain the sense of smell. This technique relies on memory and repetitive exposure to specific scents to restore olfactory function. Recently, the olfactory cell types in the upper nasal cavity have been identified as the most vulnerable to infection by SARS‐CoV‐2 [3, 26, 92]. Surprisingly, sensory neurons that detect and transmit the sense of smell to the brain are not among the vulnerable cell types [10, 19, 36, 42, 87].

Although the role of herbal medicines with antiviral and antioxidant activities has been examined, the potential therapeutic components of plant‐derived natural antioxidants in the regeneration of olfactory epithelium and continuous nerve cell renewal after an injury have not been well‐investigated for long COVID symptoms, particularly in LGBTQIA+ [97]. Clinical trials are currently examining various antioxidant compounds, like Previfenon and Melatonin, to determine their impact on the neurological functions of patients experiencing loss of smell after COVID‐19. However, the mechanism of action of these neurodegenerative agents on the brain metabolism remains unclear, particularly in the most vulnerable marginalized communities such as LGBTQIA+ [98]. Further research is needed to explore the role of natural products in addressing neurological and cognitive health issues, especially for the LGBTQIA+ community struggling with smell deficiency.

## 4 Conclusions and Outlook

To summarize, the significant effects of long COVID on public health and marginalized groups underscore the critical need for adopting Systems Thinking models. Furthermore, this article advocates for a unified approach among experts to promote interdisciplinary, collaborative research and to establish robust support strategies for COVID‐19 survivors throughout various communities. This includes a specific focus on addressing the scientific, social, and behavioral hurdles encountered by LGBTQIA+ and low‐income populations.

Building on the insights gained from employing MBST in healthcare, future initiatives should focus on tailoring this model to meet the specific needs of various marginalized groups to develop targeted interventions addressing unique health disparities in this population. Additionally, exploring the efficacy of natural product formulations in managing symptoms of chronic conditions like long COVID presents a promising area of research. These formulations should be tested for their safety, effectiveness, and potential interactions with conventional treatments.

Additionally, exploring the efficacy of natural product formulations in managing symptoms of chronic conditions like long COVID presents a promising area of research. These formulations should be tested for their safety, effectiveness, and potential interactions with conventional treatments in marginalized populations.

Expanding collaborative efforts between healthcare providers, researchers, patient advocacy groups, and the affected communities is crucial. Such collaborations can enhance the development of culturally sensitive healthcare models that understand the complex socioeconomic and health dynamics and actively involve the community in their health strategies. By integrating community feedback and clinical research, healthcare systems can innovate and adapt more effectively to meet diverse patient needs. These steps will pave the way for a more inclusive and responsive healthcare system that leverages the full potential of MBST in improving LGBQTA+ patients’ outcomes and advancing public health.

For future work, integrating portable bedside very low‐field magnetic resonance imaging (MRI) and/or positron emission tomography (PET) imaging within a Systems Thinking framework presents a promising approach to addressing public health challenges, especially in managing long‐term COVID‐19 and its sensory impacts within marginalized communities.

Although our conclusions and strategies may vary, we advocate for initiating small‐scale pilot research trials exploring a wide array of treatment possibilities for all patients, especially those from communities disproportionately affected by health disparities linked to race, gender, and sexuality. This approach aims to foster a healthier society and improve global living conditions.

## Author Contributions

This manuscript was collaboratively written, incorporating the contributions of all authors equally, each of whom has reviewed and approved the final version. B.A. played a significant role in the project, taking on responsibilities in conceptualization, investigation, writing the original draft and review/editing, formal analysis, supervision, and managing resources. These contributions span several crucial aspects of the project, ensuring thorough oversight and execution. B.A. designed the Systems Dynamic, CLD, and MBST framework and crafted a detailed MATLAB plan to study olfactory dysfunction in long COVID patients within marginalized communities. J.M.W. contributed to the investigation and conceptualization and was involved in both the original draft writing and the review and editing processes. N.B.‐Y. also played roles in conceptualization and investigation and was engaged in writing the original draft as well as reviewing and editing. H.L. participated in conceptualization, investigation, and the writing processes (original draft and review/editing). J.H.S.’s comprehensive role included conceptualization, investigation, writing the original draft, review and editing, formal analysis, project administration, supervision, and managing resources.

## Ethics Statement

This work did not involve human subjects, and therefore, no Institutional Review Board (IRB) applications or approval notices from the American Political Science Association (APSA)’s Principles and Guidance for Human Subjects Research and the American Association of Public Opinion Research (AAPOR)’s Code of Ethics are required.

## Consent

No living participants for the participation in the current manuscript are to be declared.

## Conflicts of Interest

The authors declare no conflicts of interest.

## Data Availability

No datasets were generated or analyzed in this study; thus, data sharing is not applicable.
